# Derivation of a simple risk calculator for predicting clinical worsening in patients with pulmonary hypertension due to interstitial lung disease

**DOI:** 10.1016/j.jhlto.2025.100206

**Published:** 2025-01-07

**Authors:** K. El-Kersh, R. Bag, N. Bhatt, C. King, A. Waxman, F. Rischard, H. Kim, D. Cella, E. Shen, SD Nathan

**Affiliations:** a University of Arizona College of Medicine, Phoenix; b Mayo Clinic; c The Ohio State University Medical Center; d Inova Fairfax Hospital; e Brigham and Women’s Hospital; f University of Arizona College of Medicine, Tucson; g North Carolina State University; h United Therapeutics Corporation

**Keywords:** Pulmonary hypertension, Interstitial lung disease, Risk assessment

## Abstract

**Background:**

Pulmonary hypertension due to interstitial lung disease (ILD-PH) portends very poor clinical outcomes, with a median survival time of 1.5 to 2 years. Currently, there is no tool to assess the risk of clinical worsening in patients with ILD-PH. Our aim was to derive a simple and practical risk calculator that could be used to predict risk of clinical worsening in patients with ILD-PH.

**Methods:**

The INCREASE study was a 16-week study that evaluated inhaled treprostinil in patients with ILD-PH. Baseline data from patients who were randomized to the placebo arm (n=163) and thus untreated with any approved pulmonary artery vasodilators were used to derive a risk calculator. The endpoint of interest was the time to clinical worsening. Stepwise regression, Harrell’s c-index, and clinician input were used to derive 2 multivariable Cox PH models from a set of candidate variables. The models were then simplified by applying a point-scoring system to the predictors and refitting with total point score as the covariate. Total point scores were grouped into 3 risk strata (lower, intermediate, and higher).

**Results:**

Two versions of a risk calculator were derived. The first was a non-invasive risk calculator which included NT-proBNP and FVC%/DLCO%, and a second adds cardiac index, an invasive parameter, to the above two parameters. For the total point score models, the estimated c-indices were 0.703 (95% CI: 0.635, 0.783) and 0.683 (95% CI: 0.612, 0.761) for the invasive and non-invasive model, respectively.

**Conclusion:**

These two risk calculators provide a simple way to risk stratify ILD-PH patients with clinically useful discrimination. The calculators are easy to employ in clinical practice, since they utilize assessments commonly collected in the care of patients with ILD-PH. Moreover, the calculators can provide clinicians with important prognostic information which can be used to reinforce the benefits of therapy. The risk calculators may also find utility as part of the composite allocation score of ILD-PH patients listed for lung transplant. Future research in this area could include incorporating longer-term outcomes as well as validating the risk models in a separate patient population.

## Introduction

Pulmonary hypertension (PH) commonly complicates the course of interstitial lung disease (ILD), a heterogeneous group of disease characterized by abnormalities in the lung interstitium resulting in impaired gas exchange and lung function. The prognosis associated with pulmonary hypertension due to interstitial lung disease (ILD-PH) is dismal, with a median survival of 1.5 to 2 years which is significantly shorter than survival with ILD alone.^1^, ^2^, ^3^ Given these poor outcomes, concomitant PH in setting of ILD warrants consideration of listing for lung transplantation.^4^

Risk assessment has emerged as an objective approach to evaluating patients with pulmonary arterial hypertension (PAH) and is now recommended by the ESC/ERS treatment guidelines.^5^ Risk assessment for patients with PAH invokes the risk of mortality within 1-year, and patients’ risk categorization at diagnosis and follow-up not only informs about prognosis, but also helps guide treatment decisions.^5^ Several tools are available to PAH clinicians such as the REVEAL 2.0 risk score and the European risk assessment methodology.^6^, ^7^

At present, there is no similar clinical tool to assess the risk of worse clinical outcomes in patients with ILD-PH. Our aim was to derive a simple and practical risk calculator that could be used to predict risk of clinical worsening in patients with ILD-PH.

## Methods

The INCREASE study was a 16-week study that evaluated inhaled treprostinil in patients with ILD-PH.^8^ Data from patients who were randomized to the placebo arm (n=163) and thus untreated with any approved pulmonary artery vasodilators were used to derive a risk calculator. Patients with missing baseline data were excluded. Candidate baseline variables included 6-minute walking distance (6MWD), pulmonary vascular resistance (PVR), mean pulmonary arterial pressure (mPAP), N-terminal pro b-type natriuretic peptide (NT-proBNP), percent predicted diffusing capacity of the lungs for carbon monoxide (%DLCO), percent predicted forced vital capacity (%FVC), supplemental oxygen flow rate at rest, cardiac output (CO), cardiac index (CI), oxygen saturation (SpO_2_) nadir during 6-minute walk test, resting SpO_2_, %FVC/%DLCO ratio, ratio of SpO_2_ to fraction of inspired oxygen (SpO_2_/FiO_2_), sex, and GAP stage.^9^ The outcome of interest was time to clinical worsening, defined in the INCREASE study as time from randomization to first event of cardiopulmonary hospitalization, >15% decrease in 6MWD, lung transplantation, or death. Univariate Cox proportional hazards models were fit to assess the relationship between each candidate variable and the outcome.

Multivariable Cox models were derived adjusting for different combinations of baseline characteristics using stepwise regression (a variable selection technique), Harrell’s c-index,^10^ and clinician input (KEK, SDN). These models were ‘continuous’ models, meaning the predictors were all continuous variables. The baseline variables selected for the continuous models (NT-proBNP, FVC/DLCO [% predicted], and cardiac index) were turned into categorical variables by grouping their respective ranges of values into three clinically meaningful bins based on clinician input (KEK, SDN). Two new Cox models were fit (one for the invasive model that included cardiac index and one for the non-invasive model excluding cardiac index), adjusting for the categorical versions of the selected baseline variables. For both models, points were assigned to each level of the categorical variables by scaling the corresponding regression estimate and rounding to the nearest integer. The total point score, for a given observation, could then be calculated by adding together the points scored on each variable in the model. Final Cox models (invasive and non-invasive) were fit, adjusting for total point score as the only variable.

Total point scores were grouped into 3 strata (lower, intermediate, higher). Estimating the c-index on the same data used to derive the model may be biased (eg, may be overly optimistic in the estimate of the model’s performance) so to reduce this bias, model discrimination was assessed by optimism-corrected estimates of the c-index, for both the invasive and non-invasive point score calculators.

As an exploratory analysis, we applied the REVEAL Lite 2 methodology to a subset of the placebo arm of the INCREASE trial to evaluate the performance (assessed via c-index) of variables and cut points in REVEAL Lite 2 in predicting clinical worsening over 16 weeks.^11^ WHO functional class was not collected in the INCREASE trial and was unavailable for calculating the REVEAL Lite 2 risk score.

## Results

Baseline patient characteristics of the derivation cohort are shown in Table 1. Among all patients included in the multivariable models, 44 patients experienced a clinical worsening event over the 16 weeks of the study. Variables found to be predictive (at α = 0.05) of clinical worsening based on univariate Cox proportional hazards analyses included: 6MWD, PVR, NT-proBNP (raw and log-scale), %DLCO, supplemental oxygen flow rate at rest, CO, CI, %FVC/%DLCO, and SpO_2_/FiO_2_. Table 2 summarizes results from univariate Cox proportional hazards models for each candidate variable.

**Table 1:** Summary of Candidate Baseline Variables

Baseline variable	Derivation cohort (n = 163*)
Median (Q1,Q3) 6MWD (m)	260.0 (195.0, 323.0)
Median (Q1,Q3) PVR (WU)	5.1 (4.2, 7.2)
Median (Q1,Q3) PAPm (mmHg)	35.0 (30.0, 40.0)
Median (Q1,Q3) NT-proBNP (pmol/L)	49.7 (22.9, 247.0)
Median (Q1,Q3) DLco%	26.0 (20.0, 33.0)
Median (Q1,Q3) FVC%	61.0 (49.0, 76.0)
Median (Q1,Q3) Supplemental Oxygen Flow Rate at Rest (L/min)	2.0 (0.0, 4.0)
Median (Q1,Q3) CO (L/min)	4.5 (3.9, 5.4)
Median (Q1,Q3) CI (L/min^2^)	2.4 (2.0, 2.7)
Median (Q1,Q3) SPO_2_ during Walk (%)	78.0 (74.0, 84.0)
Median (Q1,Q3) SPO_2_ Pre-Walk (%)	96.0 (92.0, 98.0)
Median (Q1,Q3) FVC%/DLco%	2.4 (1.8, 3.2)
Median (Q1,Q3) SPO_2_/FiO_2_	319.6 (265.6, 428.6)
Frequency (%) Sex	
Male	95 (58.3)
Female	68 (41.7)
Frequency (%) GAP stage	
Stage 1	23 (15.1)
Stage 2	84 (55.3)
Stage 3	45 (29.6)

* Variables with different n: NT-proBNP (n = 158), DLco% (n = 153), FVC% (n = 161), CI (n = 162), SPO_2_ during Walk (n = 153), SPO_2_ Pre-Walk (n = 162), FVC%/DLco% (n = 152), SPO_2_/FiO_2_ (n = 162), GAP stage (n = 152)

**Table 2:** Univariate Cox Proportional Hazards Model Results

Baseline variable	n	Hazard ratio (95% CI)	P-value
6MWD	163	0.995 (0.992, 0.998)	0.002
PVR	163	1.166 (1.078, 1.262)	<0.001
Mean Pulmonary Artery Pressure (PAPm)	163	1.024 (0.992, 1.057)	0.141
NT-proBNP	158	1.001 (1.001, 1.002)	<0.001
Log(NT-proBNP)	158	1.469 (1.23, 1.755)	<0.001
DLCO%	153	0.949 (0.920, 0.980)	0.001
FVC%	161	1.000 (0.986, 1.013)	0.963
Supplemental Oxygen Flow Rate at Rest	163	1.169 (1.028, 1.330)	0.017
CO	163	0.662 (0.511, 0.857)	0.002
CI	162	0.545 (0.324, 0.918)	0.022
SpO_2_ during Walk	153	0.985 (0.954, 1.017)	0.354
SpO_2_ Pre-Walk	162	0.986 (0.938, 1.036)	0.565
FVC%/DLCO%	152	1.187 (1.073, 1.312)	0.001
SpO_2_/FiO_2_	162	0.996 (0.993, 0.999)	0.012
Sex (ref = male)	163	0.817 (0.472, 1.413)	0.469
GAP stage*	152		0.469
Stage 1		0.531 (0.193, 1.461)	
Stage 2		0.880 (0.468, 1.655)	

*Hazard ratios for GAP stage use Stage 3 as the reference

There were 147 placebo arm patients with complete data for fitting the noninvasive multivariable, continuous Cox proportional hazards model, which included log(NT-proBNP) and FVC%/DLCO%, and there were 146 placebo arm patients with complete data for fitting the invasive multivariable model, which adds cardiac index (invasive parameter) to the parameters included in the noninvasive model. The multivariable, continuous Cox proportional hazards model results are presented in Table 3. Table 4 includes a summary of baseline characteristics for the subset of the derivation cohort included in the final models, as well as baseline summaries by clinical worsening subgroup (ie, subgroup of patients who experienced a clinical worsening event vs. subgroup who did not).

**Table 3:** Multivariable Continuous Cox Proportional Hazards Model Results

	Invasive model (n = 146)		Non-invasive model (n = 147)	
Baseline variable	Hazard ratio (95% CI)	P-value	Hazard ratio (95% CI)	P-value
Log(NT-proBNP)	1.435 (1.174, 1.753)	<0.001	1.500 (1.235, 1.822)	<0.001
FVC%/DLco%	1.182 (1.057, 1.322)	0.003	1.165 (1.037, 1.310)	0.010
CI	0.542 (0.299, 0.981)	0.043	--	--

**Table 4:** Baseline Demographics by Clinical Worsening Status, Among Placebo Patients Included in Final Non-invasive Risk Model

Baseline variable	Patients included in final non-invasive risk model who had a CW event (n = 44^a^)	Patients included in final non-invasive risk model who did not have a CW event (n = 103^b^)	Overall (n = 147^c^)
Median (Q1,Q3) 6MWD	232.0 (152.5, 296.5)	268.0 (224.0, 333.0)	260.0 (198.0, 322.0)
Median (Q1,Q3) PVR	5.6 (4.5, 8.5)	4.8 (4.0, 6.9)	5.0 (4.2, 7.2)
Median (Q1,Q3) PAPm	35.0 (31.5, 42.5)	34.0 (30.0, 40.0)	35.0 (30.0, 40.0)
Median (Q1,Q3) NT-proBNP	91.7 (36.1, 476.5)	37.6 (16.3, 136.0)	49.6 (22.3, 239.0)
Median (Q1,Q3) DLco%	22.0 (18.0, 27.5)	28.0 (22.0, 37.0)	26.0 (20.0, 33.0)
Median (Q1,Q3) FVC%	66.0 (54.0, 78.0)	61.0 (50.0, 74.0)	61.0 (51.0, 76.0)
Median (Q1,Q3) Supplemental Oxygen Flow Rate at Rest	3.0 (2.0, 4.0)	2.0 (0.0, 4.0)	2.0 (0.0, 4.0)
Median (Q1,Q3) CO	4.1 ( 3.3, 4.9)	4.7 (4.0, 5.7)	4.5 (3.9, 5.5)
Median (Q1,Q3) CI	2.2 (1.9, 2.5)	2.4 (2.1, 2.8)	2.4 (2.0, 2.7)
Median (Q1,Q3) SPO_2_ during Walk	78.0 (73.5, 82.0)	78.5 (75.0, 85.0)	78.0 (74.0, 85.0)
Median (Q1,Q3) SPO_2_ Pre-Walk	95.0 (92.0, 98.0)	96.0 (93.0, 98.0)	96.0 (92.0, 98.0)
Median (Q1,Q3) FVC%/DLco%	2.9 (2.0, 3.7)	2.2 (1.6, 3.0)	2.3 (1.8, 3.2)
Median (Q1,Q3) SPO_2_/FiO_2_	306.3 (257.6, 350.0)	341.1 (272.2, 447.6)	328.6 (272.2, 428.6)
Frequency (%) Sex			
Male	28 (63.6)	58 (56.3)	86 (58.5)
Female	16 (36.4)	45 (43.7)	61 (41.5)
Frequency (%) GAP stage			
Stage 1	5 (11.4)	18 (17.5)	23 (15.7)
Stage 2	25 (56.8)	55 (53.4)	80 (54.4)
Stage 3	14 (31.8)	30 (29.1)	44 (29.9)

a Variables with different n: SPO_2_ during Walk (n = 40)

b Variables with different n: CI (n = 102), SPO_2_ during Walk (n = 98), SPO_2_ Pre-Walk (n = 102), SPO_2_/FiO_2_ (n = 102)

c Variables with different n: CI (n = 146), SPO_2_ during Walk (n = 138), SPO_2_ Pre-Walk (n = 146), SPO_2_/FiO_2_ (n = 146)

**Figure 1:**
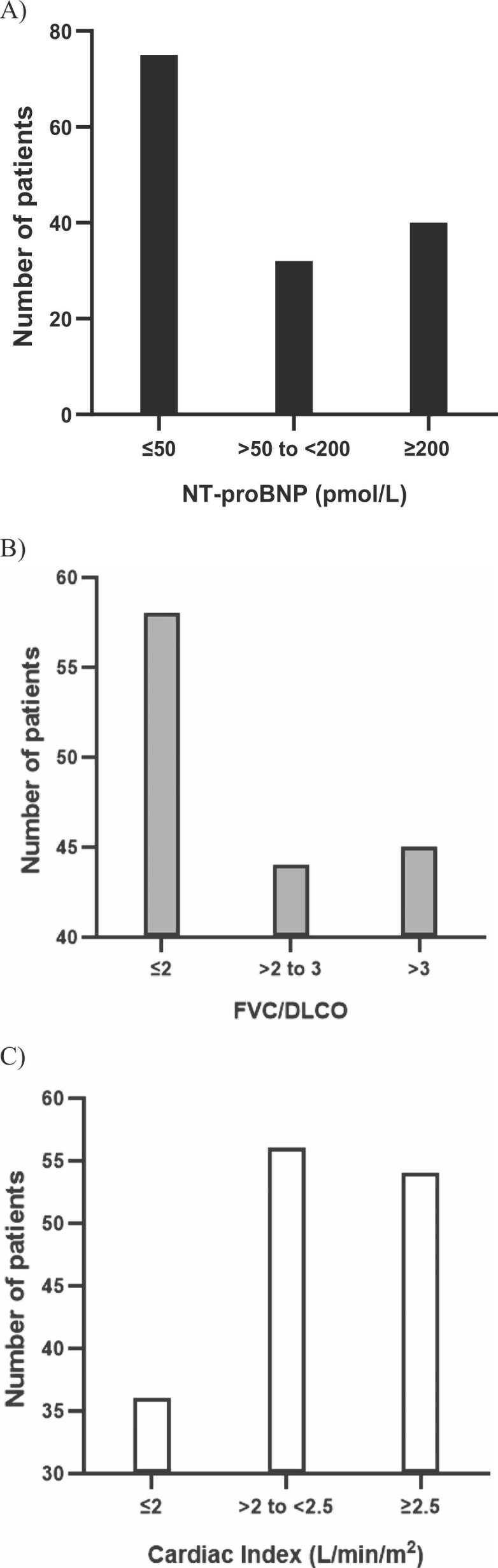
Patient distribution of variables included in final risk models. (A) Bar graph of NT-proBNP based on the n=147 patients included in the noninvasive model. (B) Bar graph of FVC%/DLCO% based on the n=147 patients included in the noninvasive model. (C) Bar graph of cardiac index based on the n=146 patients include in the invasive model.

**Figure 2:**
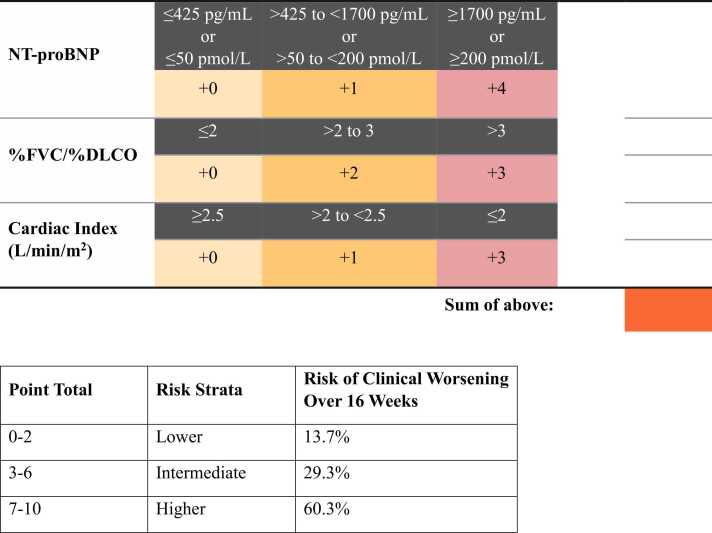
Invasive risk score calculator.

**Figure 3:**
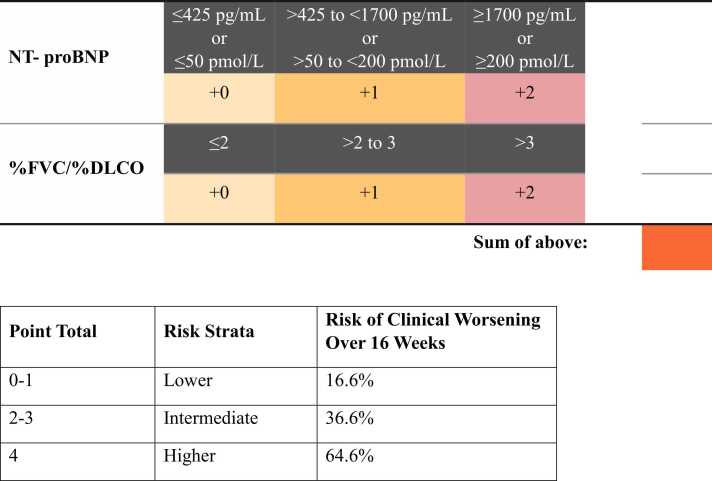
Non-invasive risk score calculator.

The cut points for fitting the categorical Cox proportional hazards models were as follows. For NT-proBNP: ≤50 pmol/L (≤425 pg/mL), >50 to <200 pmol/L (>425 to <1700 pg/mL), and ≥200 pmol/L (≥1700 pg/mL); for FVC/DLCO [% predicted]: ≤2, >2 to ≤3, and >3; and for cardiac index (L/min/m^2^): ≤2, >2 to <2.5, and ≥2.5. Bar plots of these three variables are displayed in Figure 1. For the noninvasive risk model, total scores were grouped into risk strata such that ∼40% of patients were classified as lower risk, ∼50% as intermediate risk, and 10% as higher risk. For the invasive model, the grouping was such that ∼30% of patients were classified as lower risk, ∼50% as intermediate risk, and ∼20% as higher risk. Visual summaries of the invasive and noninvasive risk calculators are shown in Figure 2, Figure 3, respectively. The first part of each figure shows how to calculate the total points for a given set of baseline parameter values. In the bottom of each figure, point scores can be mapped to a corresponding risk stratum. The model-predicted chance of a clinical worsening event by Week 16 is also displayed for each risk stratum.

The estimated c-index was 0.688 (95% CI: 0.612, 0.767) for the invasive continuous Cox model and 0.708 (95% CI: 0.628, 0.790) for the non-invasive continuous Cox model. Regarding the total point score models, the estimated c-indices were 0.703 (95% CI: 0.635, 0.783) and 0.683 (95% CI: 0.612, 0.761) for the invasive and non-invasive model, respectively.

**Figure 4:**
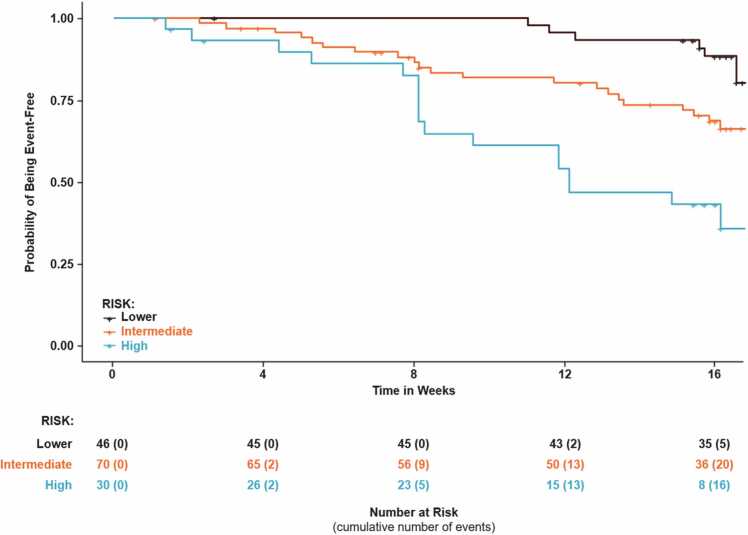
Kaplan-Meier Plot of Time to Clinical Worsening for Invasive Risk Score Calculator.

**Figure 5:**
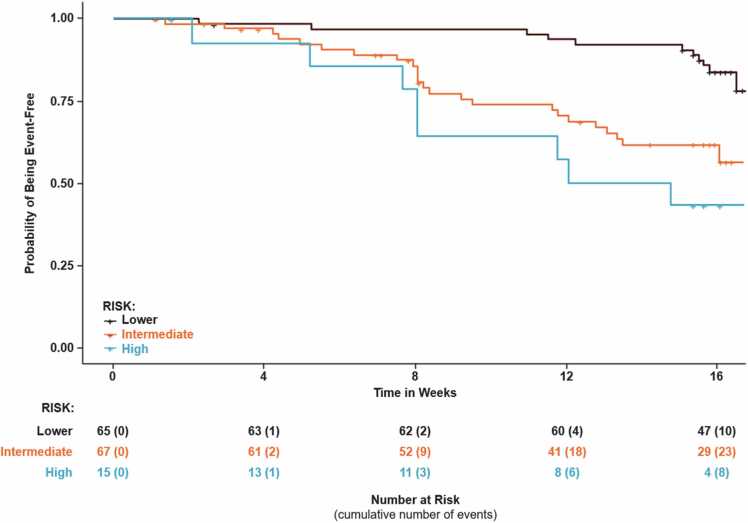
Kaplan-Meier Plot of Time to Clinical Worsening for Noninvasive Risk Score Calculator.

**Figure 6:**
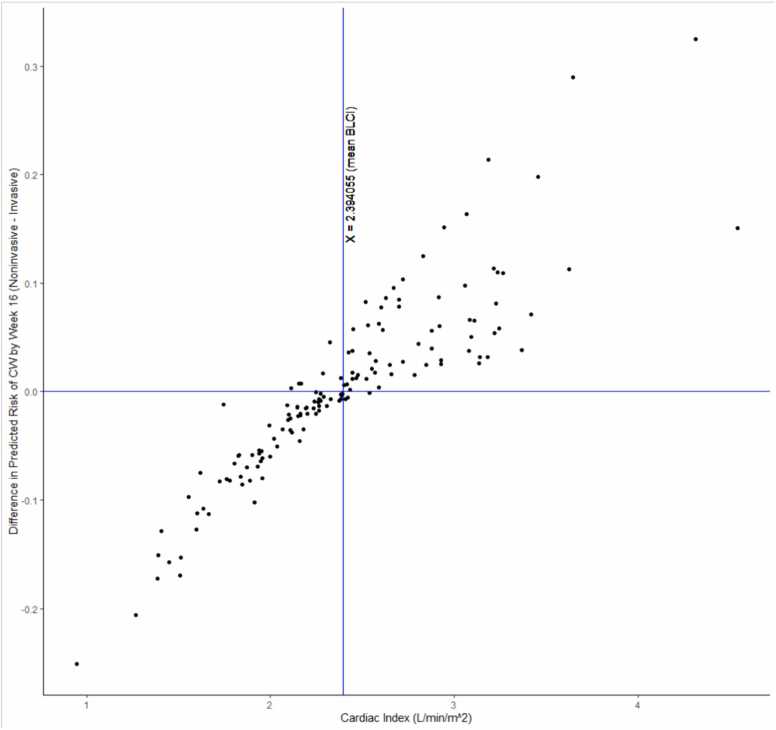
Scatterplot of difference in predicted risk of clinical worsening between the non-invasive and invasive models vs. cardiac index (L/min/m^2^).

113 (77%) patients had the same risk stratum assignment from the invasive and non-invasive calculators; 33 (23%) patients had a different risk group assignment. In all 33 cases where the risk stratum between the calculators did not match, the non-invasive risk calculator assigned a lower risk than the invasive stage. Kaplan-Meier plots of time to clinical worsening for each risk group are shown for the two risk calculators in Figure 4, Figure 5. To assess the relationship between cardiac index and the risk predicted by the invasive vs. noninvasive continuous models, the difference in predicted risk of clinical worsening over 16 weeks (noninvasive – invasive) was plotted against cardiac index for the 146 patients which the two models had in common (Figure 6).

In our exploratory analysis using the 146 placebo patients from the noninvasive model who had baseline variables required for REVEAL Lite 2.0, the REVEAL Lite 2.0 total point score model had an estimated c-index of 0.691 (95% CI: 0.61, 0.781) using clinical worsening as the outcome measure. Table 5 shows the risk of clinical worsening over 16 weeks as predicted by REVEAL Lite 2.0.

**Table 5:** Model-Predicted Risk of Clinical Worsening within 16 Weeks by Risk Strata, Based on Cox PH Model for REVEAL Lite 2 Total Point Score

Point total^a^	Risk strata	Risk of clinical worsening over 16 weeks
2 to 5	Lower	16.1%
6 to 7	Intermediate	28.3%
8 to 12	Higher	54.1%

^a^ WHO functional class was not collected in the INCREASE study so the possible score range is 2 to 12, instead of 1 to 14.

## Discussion

We describe two versions of a simple model to risk stratify ILD-PH patients for clinical worsening within 16 weeks with clinically useful discrimination. Both risk score calculators exhibit good separation between the 3 risk strata.

The non-invasive calculator performed similarly to the invasive calculator and utilizes variables that are easily collected. Interestingly, the addition of 6MWD or degree of desaturation during the 6MWT to models did not improve their performance as assessed by c-indices. The FVC/DLCO ratio provides a surrogate for the extent of parenchymal disease (FVC%) relative to the associated vasculopathy (DLCO), while the NT-proBNP is a surrogate for the associated right ventricular stress and strain. It is not surprising therefore that inclusion of the cardiac index in the invasive model impacts risk predictions even though model discrimination remains relatively similar because the invasive model captures the additional element of forward flow compromise. In particular, we noted that the further the cardiac index was from the sample mean, the greater the impact on the predicted risk of clinical worsening (Figure 6). In our analysis, in patients with a CI below the sample mean, the predicted risk tended to be underestimated by the non-invasive model. For patients with a CI above the sample mean, the predicted risk tended to be overestimated by the non-invasive model. A similar observation was noted in PAH patients who had discrepancy between their REVEAL Lite 2 risk score and REVEAL-Echo risk score.^12^ In these patients with high-risk echocardiographic features and lower REVEAL Lite 2 risk category, using REVEAL Lite 2 alone could underestimate the risk of mortality.^12^

This similarity in risk modeling between different etiologies of pulmonary hypertension is unsurprising; the cardiac index is a measure of RV function and has been shown to be an independent predictor of survival in both patients with ILD-PH and PAH.^13^, ^14^ Also, the RV dysfunction in group 3 PH can be further impacted by other afterload-independent, non-hemodynamic factors such as direct insult from hypoxia, systemic inflammation, and endothelial dysfunction in setting of the underlying ILD.^15^

In the same vein, our exploratory analysis of using REVEAL Lite 2.0 resulted in a c-index similar to the invasive and non-invasive models derived in this study. Although the REVEAL risk calculator was designed to predict mortality in patients with PAH (as opposed to clinical worsening in patients with ILD-PH, as we used the model) it is perhaps expected that the REVEAL Lite 2.0 risk calculator worked well in the INCREASE population; REVEAL includes many variables that are markers of vascular disease and exercise limitation that are observed in patients with both PAH and ILD-PH. Without the raw data from the REVEAL registry, we are unable to determine which of the shared parameters between our models and REVEAL may be “driving” the similar c-indices, but we postulate that the NT-proBNP may have an influence as this biomarker is strongly correlated with prognosis in both PAH and ILD.^5^, ^16^

Risk assessment in this patient population is an area of active research, including the risk of patients with ILD developing PH as well as the risk of death and poor clinical outcomes once PH manifests.^17^, ^18^, ^19^, ^20^, ^21^ Other PAH risk calculators have been experimentally applied to patients with ILD-PH to determine their predictive strengths. Yogeswaran and colleagues applied a truncated form of the ESC/ERS risk assessment to a group of 185 patients with severe ILD-PH and found that 5-year transplant-free survival of lower-, intermediate-, and high-risk groups were 43%, 15%, and 4%.^22^ The variables used to risk stratify patients in this analysis included functional class, 6-minute walking distance (6MWD), brain natriuretic peptide (BNP), right atrial area, pericardial effusion, right atrial pressure, cardiac index, and mixed venous oxygen saturation. In contrast to the risk models we derived, FVC and DLCO were not found to independently correlate to mortality. Their study also differs from ours in that it only included a more “vascular phenotype” of severe ILD-PH with mPAP ≥35 mmHg (or ≥25 mmHg with cardiac index <2.0 liter/min/m^2^), and the outcome measure was different. However, both this prior study and this present analysis include BNP (or NT-proBNP), suggesting the significance of right heart strain on clinical outcomes in patients with ILD-PH, reinforcing the notion that markers of vascular disease may drive prognosis in this patient population.

It has previously been well-documented that PH complicating the course of fibrotic ILD is associated with worse outcomes. However, there is a paucity of data evaluating risk within patients with established ILD-PH and this is where our model(s) are unique. Similar, we posit that it is important to incorporate markers for PH in the general prognostication of patients with ILD. Indeed, this might be why the recently described DOGAP score performs better than the original GAP score in discerning outcomes in patients with IPF. Specifically, the DOGAP incorporates oxygen use and 6MWD which might both be surrogates for an underlying vasculopathy.^23^ These markers of PH may also have utility when used in conjunction with the lung Composite Allocation Score (CAS), which currently includes the mean pulmonary artery pressure as its sole measure of vascular disease. Our data provides a rationale for including both the DLCO and the NT-proBNP as required variables when patients are listed for lung transplantation.

Our study is limited by the relatively short follow-up time of 16 weeks used in the INCREASE study. It is unknown whether the risk calculators we derived maintain their accuracy over longer time periods, or what effect vasoactive treatment may have on risk scores given that the models were calculated using an untreated placebo group. Additional research is needed to elucidate whether therapy-related reductions in risk score in patients with ILD-PH is associated with improvements in clinical outcomes. In addition, our model(s) might be useful in risk stratifying subjects in future clinical trials of ILD-PH and could possibly be employed as composite endpoints for any such future trials. It is important to note that these risk calculators should not be used to dictate therapy, since it is well established that even patients with mild ILD-PH have poor outcomes and benefit from therapy.^24^ Nonetheless, these prediction equations do help clinicians in providing important prognostic information which can be used to reinforce the benefits of therapy. Furthermore, these prediction models could be explored to evaluate if they add more granularity to lung CAS in ILD patients to prioritize sicker patients with higher risk of worsening/ mortality. These models highlight the notion that once PH develops in patients with ILD, it is the primary determinant of prognosis as all the markers in both models are markers of pulmonary vascular disease severity rather than ILD. For instance, percent predicted FVC and DLCO were considered independently as candidate variables for the models, along with their ratio, but it was the ratio—which is often elevated in patients with PH and lung disease due to systemic sclerosis—that was selected by the variable selection procedure.^25^

In conclusion, we describe two risk calculators to gauge the risk of clinical worsening in patients with ILD-PH. These models are simple to use and employ in clinical practice since they incorporate variables that are commonly collected. Our description lays the foundation for future research to validate our models in a separate validation cohort, as well as in other populations and over a longer period of time. Incorporation of the DLCO and NT-proBNP as part of lung transplant listing criteria could be of benefit in further refining the CAS system and would be a valuable future resource to validate and refine our models. Whether our non-invasive model might predict outcomes in patients without documented PH is another open question for future research.

## Author Contributions

The authors confirm contribution to the paper as follows: study conception and design: KEK, DC, ES, SDN. Data acquisition: KEK, RB, NB, CK, ABW, FR, SDN. Statistical analysis: HK, DC. Interpretation of results: all authors. Draft manuscript preparation: KEK, DC, ES, SDN. All authors reviewed the results and approved the final version of the manuscript.

## Conflict of Interests

KEK consults for United Therapeutics Corp., Merck, and Johnson & Johnson. RB has no relevant disclosures. NB has received consulting fees from United Therapeutics Corp. CK has received consulting fees and is on the speaker bureau for United Therapeutics Corp. ABW has received grant support or consulting fees from AI Therapeutics, ARIA-CV, Acceleron/Merck, Janssen Pharmaceuticals, and has participated on a data and safety monitoring board for Insmed. FR is a consultant for Acceleron and United Therapeutics and is on a steering committee for Acceleron, Ismed, United Therapeutics, Bayer, Acceleron, Janssen and AADI. HK has no relevant disclosures. DC and ES are employees of United Therapeutics. SDN has received consulting fees from Bellerophon Therapeutics, United Therapeutics, Merck, Roivant, Insmed, Analyn Pharma, Boehringer-Ingelheim, PureTech, and Excalibur; is a speaker for United Therapeutics and Boehringer-Ingelheim; is on the board for Gossamer Bio; and has participated on a data and safety monitoring board for Horizon Pharma.

## Disclosures

KEK consults for United Therapeutics Corp., Merck, and Johnson & Johnson. RB has no relevant disclosures. NB has received consulting fees from United Therapeutics Corp. CK has received consulting fees and is on the speaker bureau for United Therapeutics Corp. ABW has received grant support or consulting fees from AI Therapeutics, ARIA-CV, Acceleron/Merck, Janssen Pharmaceuticals, and has participated on a data and safety monitoring board for Insmed. FR is a consultant for Acceleron and United Therapeutics and is on a steering committee for Acceleron, Ismed, United Therapeutics, Bayer, Acceleron, Janssen and AADI. HK has no relevant disclosures. DC and ES are employees of United Therapeutics. SDN has received consulting fees from Bellerophon Therapeutics, United Therapeutics, Merck, Roivant, Insmed, Analyn Pharma, Boehringer-Ingelheim, PureTech, and Excalibur; is a speaker for United Therapeutics and Boehringer-Ingelheim; is on the board for Gossamer Bio; and has participated on a data and safety monitoring board for Horizon Pharma.

## Funding

Journal submission fees were supported by United Therapeutics Corporation. The INCREASE study was sponsored by United Therapeutics Corporation.
